# “I worry if I will have food tomorrow”: a study on food insecurity among asylum seekers living in Norway

**DOI:** 10.1186/s12889-019-6827-9

**Published:** 2019-05-17

**Authors:** Sigrun Henjum, Marianne Sandsmark Morseth, Charles D. Arnold, Dawid Mauno, Laura Terragni

**Affiliations:** 1 Department of Nursing and Health Promotion, Faculty of Health Sciences, Oslo Metropolitan University, 0130 Oslo, Norway; 2 Department of Nutrition, University of California, Davis, CA USA

**Keywords:** Asylum seekers, Food security, Child hunger, Adult hunger, Household food insecurity, Reception centers, Norway

## Abstract

**Background:**

High prevalence of food insecurity has been observed among asylum seekers resettled in high-income countries. Economic constraints, lack of knowledge about new foods, difficulties with shopping, challenges with language, as well as problems complying with various religious food rules are associated with the occurrence and severity of food insecurity. However, no data on food security among asylum seekers in Norway currently exist. Thus, the aim of the study was to assess food security among asylum seekers living in Norwegian reception centers.

**Methods:**

Using convenience sampling, we selected eight reception centers in the southeastern part of Norway and included 205 asylum seekers, including 41 families with children < 18 years of age. We measured food security using the 10-item version of the Radimer/Cornell Hunger and Food Insecurity Scale. Food insecure participants were divided into three groups: food insecurity without hunger, food insecurity with hunger, or food insecurity with child hunger. Using logistic regression models, we analyzed the association between food insecurity status and socioeconomic variables.

**Results:**

Seven percent of the participants were categorized as food secure and 93% as food insecure, of whom 11% were food insecure without hunger, 78% were food insecure with hunger, and 4% were food insecure with child hunger. Among the families with children, 20% (8 of 41) experienced child hunger. For the participants experiencing food insecurity with hunger, 44% reported that they were hungry often, and among families with children, 14% reported that despite being aware of the child’s hunger, they did not have the resources/money to buy more food. In logistic regression models, men had higher odds of experiencing adult food insecurity with hunger than women, OR (95% CI): 4.08 (2.04, 8.16). A reduction in monthly budget by 100 euros increased the odds of experiencing adult food in-security with hunger by 1.37 times OR (95% CI), 1.37 (1.16, 1.61).

**Conclusions:**

The prevalence of food insecurity among asylum seekers in Norway was high, in contrast to low prevalence of food insecurity in the Norwegian population. Asylum seekers are a particularly vulnerable group and initiatives to ameliorate the opportunities for an adequate diet are of the outmost importance.

## Background

Food security refers to the ability of individuals, households, and communities to acquire appropriate and nutritious food on a regular and reliable basis, using socially acceptable means [1]. A high prevalence of food insecurity has been observed among asylum seekers resettled in high-income countries [2–4]. Economic constraints, lack of knowledge about new foods, difficulties with shopping, language challenges, and problems complying with various religious food rules are associated with the occurrence and severity of food insecurity [2, 3, 5]. Skipping meals and developing new eating habits based on a strategy to survive on as little money as possible has been reported [6]. A study conducted in Sweden showed that asylum seekers’ food needs are often not met and that consequently, their intake may be insufficient from a nutritional standpoint [7]. Further, a report on unaccompanied minor refugees showed that residents in Norwegian asylum centers tend to refrain from buying nutritious (more expensive) foods [8].

The number of asylum seekers heading to Norway fluctuates depending on emergencies and changes in asylum policies [9]. In the period from 2012 to 2014, approximately 10,000–12,000 asylum seekers arrived in Norway, while in 2015, the number increased to 31,000, only to fall dramatically in 2017, when the number was 3560 [10]. In January 2017, when this study started, there were 12,676 asylum seekers residing in Norwegian reception centers. Upon arriving, asylum seekers live in transit centers in order to go through a first health check and interview with the Norwegian Directorate of Immigration for their asylum application. Subsequently, they are transferred to ordinary reception centers while they wait for the results of their application. The centers are run by municipalities, private actors, or non-governmental institutions under the Norwegian Directorate of Immigration’s directives. The directorate’s regulations specify that the centers need to meet the criteria for adequate—but simple and essential—housing facilities. While waiting for a decision about their application, asylum seekers are not allowed to work, but they receive a public subsidy from Norwegian authorities, which is aimed at covering all expenses besides accommodation [11]. In 2017, the amount was about 250 euros. People who have had their applications turned down and decide to appeal see their monthly allowance reduced to 190 euros.

Several studies have investigated the living and health conditions of asylum seekers in Norway, and some of these suggested that the opportunities for having adequate diets could be precarious [8, 12, 13]. However, food and meals at asylum reception centers and food security among asylum seekers in Norway have not been explicitly investigated. The definition of food security used in this study encompasses aspects related to being worried about not having enough food, having to compromise the quality of the diet, experiencing hunger and, finally, knowing that your children are hungry [14]. Learning more about food security among asylum seekers living in Norwegian reception centers could contribute to the development and implementation of policies and actions promoting adequate diets, as well as the prevention of nutritional deficiencies and lifestyle-related diseases.

## Methods

### Sample selection and research design

In this cross-sectional study, we selected eight asylum reception centers in the southeastern part of Norway through convenience sampling and included 205 asylum seekers. The participants consisted of adult men and women > 18 years of age who lived in ordinary reception centers with self-catering. Residents had to be able to answer questions from an interview-based questionnaire. Fieldworkers speaking the most common languages of the residents in the selected asylum centers (Arabic, Somali, Dari, and Tigrinya in addition to Norwegian and English) were employed in the project. We only included participants who spoke these languages. The data collection was performed from 31 January to 25 August 2017; however, no data was collected during Ramadan (June). The asylum reception centers were selected based on their typology (centralized: residents living in one main building or decentralized: residents living in separate apartments) and location around the city center (urban) or outside the city center (rural, meaning the distance to the nearest grocery store was more than four kilometers). Half the reception centers were located in urban and half in rural areas.

### Questionnaire

The questionnaire consisted of four parts: the first part contained 12 questions on socioeconomic status, the second part was a 24-h dietary recall, the third part contained 19 questions on food skills, and the fourth part had 10 questions on food security. It was conducted as an interview between the resident and the field worker. The field workers, who received training in interview techniques from the project managers, translated the questionnaire from English into Arabic, Somali, and Dari. The project managers created the questionnaire based on already validated questionnaires, but some changes were made after pilot testing and discussions with field workers to adapt it to the conditions of asylum seekers residing in Norway. In this paper we present data on food security, in addition to background characteristics of the asylum seekers (Table 1). The variable monthly budget refers to self-reported monthly budget in euro, which includes both the public subsidy from the Norwegian authorities for each family member and addition to income from other sources (if any). The variable months in Norway refers to months since arrival to Norway. Country of origin refers to the country of origin of the asylum seeker and the four most common countries are presented in Table 1. In addition the variable “being able to cook most dishes without recipe” from the food skills questionnaire is presented in this paper.

**Table 1:** Asylum seekers’ background characteristics by food insecurity levels (*n* = 205)

	Food secure	Food insecure			Total n
	*n* (%)	Food insecure without hunger *n* (%)	Food insecure with hunger *n* (%)	Food insecure with child hunger *n* (%)	
Age in years^a^	35 (13)	29 (9)	31 (10)	33 (3)	31 (10)
Gender					
Female	9 (12)	15 (20)	46 (62)	4 (5)	74
Male	6 (5)	7 (5)	114 (87)	4 (3)	131
Country of origin^b^					
Syria	4 (8)	3 (6)	42 (81)	3 (6)	52
Eritrea	5 (14)	6 (17)	23 (64)	2 (6)	36
Somalia	1 (5)	1 (5)	20 (91)	–	22
Iraq	1 (5)	2 (9)	18 (82)	1 (5)	22
Other	4 (6)	10 (14)	59 (78)	2 (3)	73
Application status					
Submitted	3 (6)	8 (16)	51 (76)	1 (2)	49
Rejected	4 (7)	4 (7)	50 (82)	3 (5)	61
Granted	8 (10)	10 (12)	59 (73)	4 (5)	81
Months in Norway^a^	27 (31)	25 (25)	30 (32)	28 (29)	28 (31)
Reception center					
Urban area	8 (7)	16 (13)	91 (76)	5 (4)	85
Rural area	7 (8)	6 (7)	69 (81)	3 (4)	120
Marital status					
Married	7 (9)	9 (11)	57 (72)	6 (8)	79
Single	3 (3)	12(13)	80 (84)	–	95
Other	5 (16)	1 (3)	23 (74)	2 (7)	31
Education					
None	1 (3)	3 (8)	35 (90)	–	39
0–12 years	10 (11)	13 (14)	66 (70)	5 (5)	94
Higher education	4 (6)	6 (9)	58 (82)	3 (4)	71
Employed before					
Yes	11 (8)	12 (9)	106 (78)	7 (5)	67
No	4 (6)	10 (15)	52 (78)	1 (2)	136
Monthly budget (euros)^a^	446 (320)	338 (191)	290 (160)	586 (217)	320 (193)
Food donations^c^	1 (7)	6 (27)	13 (8)	1 (13)	21 (10)
Cook without recipe	11 (79)	15 (68)	80 (50)	6 (75)	112 (50)
Food insecurity levels	15 (7)	22 (11)	160 (78)	8 (4)	205

^a^Mean (SD); ^b^Only the four most common countries are listed; ^c^From grocery stores or NGOs

### Measurement of food security

Food insecurity was assessed using the 10-item version of the Radimer/Cornell Hunger and Food Insecurity Scale [14, 15]. The scale includes qualitative and quantitative aspects of food security and consists of 10 questions divided into three categories: food insecurity without hunger (4 questions), food insecurity with hunger (4 questions), and food insecurity with child hunger (2 questions) (Table 2). Each question had three possible answers: “not true,” “sometimes true,” or “often true.” Participants were asked to refer to the last 12 months while living in reception centers. They were categorized as food secure if they responded “not true” to all questions. If the participants had at least one response of “sometimes/often” to questions 1–4 and no “not true” responses for questions 5–8, they were categorized as food insecure without hunger. Participants were categorized as food insecure with hunger if at least one response to questions 5–8 was “sometimes/often”; and they were food insecure with child hunger if at least one response to questions 9 and 10 was “sometimes/often.” Participants who, in the last 12 months, worried about whether their food would run out before they got their money, ate the same thing for several days, and reported that they ran out of food without the possibility of buying more were categorized as food insecure without hunger. Participants who reported that in the last 12 months they were often hungry, ate less than they thought they should, and could not afford to eat properly were categorized as food insecure with hunger. Families who reported that their children were not eating enough or that they knew their children were sometimes hungry but they could not afford to buy more food were categorized as food insecure with child hunger.

**Table 2:** Responses to the food insecurity and hunger questions (*n* = 205)

Statements	Positive response *n* (%)^a^
Food insecurity without hunger	
1. I worry whether my food will run out before I get money to buy more.	149 (73)
2. We eat the same thing for several days because we only have a few different kinds of foods on hand and do not have money to buy more.	150 (73)
3. The food that I bought did not last; I did not have money to get more.	136 (66)
4. I ran out of the foods that I needed to put together a meal, and I did not have money to get more food.	146 (71)
Food insecurity with hunger	
5. I am often hungry, but do not eat because I cannot afford enough food.	90 (44)
6. I eat less than I think I should because I do not have enough money for food.	123 (60)
7. I cannot afford to eat properly.	157 (77)
8. I cannot give my child(ren)^b^ a balanced meal because I cannot afford that.	15 (37)
Food insecurity with child hunger^b^	
9. My child(ren) is/are not eating enough because I just cannot afford enough food.	10 (24)
10. I know my child(ren) is/are hungry sometimes, but I just cannot afford more food.	6 (15)

^a^The response categories “often and sometimes” are merged; ^b^41 families had children; The questions referred to the respondents’ experiences while living in reception centers in the last 12 months or since their arrival at the reception centers for those who had arrived less than 12 months earlier

### Ethics

The present study was approved by the Norwegian Centre for Research data and was carried out over 7 months (31 January to 25 August 2017). The participants were informed of the study purpose and assured that the study had no implications on their application status. All informants gave written or oral informed consent to participate. The study was conducted according to the guidelines in the Declaration of Helsinki.

### Statistics

The Statistical Package for the Social Sciences version 23.0 was used to analyze the data. The significance level was 0.05. Continuous data were presented as means and standard deviations if normally distributed, and as medians if not normally distributed. Logistic regression analyses were performed to assess bivariate associations between adult food insecurity (outcome variable) and selected socioeconomic variables (exposure variables). Variables included age, gender, number of children, location of reception center (urban/rural), country of origin (Afghanistan, Syria, Iraq, Iran, Ethiopia, Somalia, Eritrea, and Other), status of application (accepted vs rejected/other), education (divided into three categories: no education, primary/secondary education, and higher education), self-reported monthly budget (continuous variable in euros, including both the received allowance for each family member and income from other sources), and months in Norway (months since arrival in Norway).

## Results

Background characteristics are presented in Table 1. The mean age of the participants was 31 years and 36% were female. Twenty-five percent of the participants came from Syria, followed by Eritrea (18%), Somalia (11%), and Iraq (11%). The mean number of months lived in Norway was 28, with 40% of participants having had their application to remain in Norway granted, 30% having had their application rejected, while the remaining were waiting for an answer from the Norwegian authorities. Nineteen percent had no education and 66% had not been employed before their arrival in Norway. Seven percent of the participants were categorized as food secure and 93% as food insecure, of whom 11% were food insecure without hunger, 78% were food insecure with hunger, and 4% were food insecure with child hunger. Among the families with children (41 families), 20% experienced child hunger. Eighty-eight percent of the women reported that they were able to cook most dishes without using recipe, compared to 37% of the men. Only 11% of the men lived with children, compared to 41% of the women.

The responses to the food insecurity and hunger questions are presented in Table 2. Among the participants experiencing food insecurity without hunger, 73% reported that they often or sometimes ran out of the foods they needed to put together a meal and did not have money to get more food. In addition, 71% stated that they actually ran out of money to buy food. A large part of the respondents (73%) stated that they tend to eat the same food several days in a raw because cannot afford to buy new food. Among the participants experiencing food insecurity with hunger, 44% reported, “I am often hungry, but do not eat because I cannot afford enough food” and 60% said, “I eat less than I think I should because I do not have enough money for food.” For families with children, 37% reported that they could not give their children a balanced meal, 24% stated that their children were not eating enough due to limited economic resources, and 15% responded that they knew their children were sometimes hungry, but they could not afford more food.

In the logistic regression models (Table 3), men had 4.08 times higher odds of experiencing adult food insecurity with hunger than women (OR (95% CI): 4.08 (2.04, 8.16)). A reduction in their monthly budget by 100 euros increased the odds of experiencing adult food insecurity with hunger by 1.37 times (OR (95% CI), 1.37 (1.16, 1.61)). Concerning education, participants with no education had higher odds of experiencing adult food insecurity with hunger than participants with a higher education (OR (95% CI), 1.96 (0.59, 6.49)) and those with a primary/secondary school education had less risk of experiencing food insecurity with hunger than participants with a higher education (OR (95% CI), 0.52 (0.25, 1.11)). Those with more children had a lower odds of experiencing adult food insecurity (OR (95% CI), 0.47 (0.33, 0.68)).

**Table 3:** Logistic regression model of adult food insecurity with hunger by background characteristics

Variable	Food insecurity Unadjusted OR (95% CI)	*P*
Age	0.99 (0.96, 1.03)	0.664
Gender^a^	4.08 (2.04, 8.16)	< 0.001
Urban	0.73 (0.37, 1.44)	0.364
Country of origin^b^	–	0.182
Application granted	0.61 (0.31, 1.19)	0.147
Education^c^		0.037
Higher education	ref	
Primary/Sec	0.52 (0.25, 1.11)	
No education	1.96 (0.59, 6.49)	
Monthly budget^d^	0.73 (0.62, 0.86)	< 0.001
Months in Norway	1.00 (0.99, 1.02)	0.547
Number of children	0.47 (0.33, 0.68)	< 0.001

^a^Gender: men compared to women; ^b^Categories include Afghanistan, Eritrea, Ethiopia, Iran, Iraq, Somalia, Syria (reference), and Other. Individual estimates not shown. Omnibus *p*-value shown, pairwise comparisons were not statistically different from one another; ^c^Education divided into three categories (no education, primary/secondary education, and higher education); ^d^Per 100 euro increase in budget

## Discussion

This study has improved our knowledge of food security among asylum seekers in Norway. It is the first study on this subject in Norway and one of the first in Europe. We found that 93% of the participants reported having experienced food insecurity within the last 12 months: among adults, 11% reported food insecurity without hunger and 78% reported food insecurity with hunger. Among families with children, 20% reported child hunger.

A high proportion of the world’s refugees are resettled in high-income countries with abundant food supplies. However, several studies within the last decade have documented a high prevalence of food insecurity among refugees in high-income countries. In the United States, Hadley et al. found that 85% of Liberian refugee households were food insecure [4]; among Somali immigrants, 72% of households were food insecure [16]; for Sudanese immigrants, 37% had experienced household food insecurity and hunger [17]; and among West African refugees, 53% had experienced food insecurity [2]. Studies from Australia and the United Kingdom have also shown that food insecurity among refugees is widespread. In the United Kingdom, 100% of refugee families with children under 5 years indicated food insecurity [18], and in a sample of newly arrived refugees in Australia, 71% reported having previously run out of food [19]. Even though the prevalence of food insecurity in these other studies varies, it is comparable to our findings and shows that food insecurity is widespread among refugees in affluent countries. The comparatively low prevalence of food insecurity in the Norwegian population (3%) [20] indicates that asylum seekers are a particularly vulnerable group even in a country such as Norway with a generous welfare state system [21]. Other studies conducted in Norway have also reported poor living conditions and poverty among asylum seekers [8, 13, 22].

In the present study, monthly budget was a strong predictor of adult food insecurity. In 2017, asylum seekers in Norway received 250 euros per person per month to cover all their expenses (food, medicine, clothing, and transport). Asylum seekers whose applications were rejected had their monthly allowance reduced to 190 euros [23]. To give an indication of the purchasing power of this allowance, one adult living in Norway would need approximately 250 euros to cover their food costs only, and a Norwegian family is using 11% of their budget on food [24]. Asylum seekers are vulnerable to food insecurity in part because of their low income. Income and specific knowledge about shopping for food in a new environment are needed to access the abundant food supplies in high-income countries [3, 5, 25]. Studies of refugees resettled in the United States, the United Kingdom, and Australia suggest that limited income is an important correlate and a plausible underlying causal determinant for high levels of food insecurity among this population [17, 18, 26, 27]. These studies have also generated qualitative data suggesting that along with income and difficulty with budgeting in a new economy, limited information about shopping and cooking options may contribute to an individual’s ability to achieve and/or maintain food security [19, 25, 28]. The fact that few asylum reception centers are located in Oslo, the Norwegian capital, where most immigrants live, could also reduce the opportunities for asylum seekers to benefit from support from relatives and acquaintances.

We found that men had almost four times higher odds of experiencing adult food insecurity with hunger than women. This could be explained in part by the differences in cooking skills among men and women. Around 40% of the men reported that they were able to cook compared to 90% of the women. Differences in being able to prepare a simple meal between men and women have also been reported in other studies [29]. This can reflect cultural differences in gender roles in the country of origin where women traditionally have the responsibility of preparing meals for the family, and male relatives and men in general might not consider it appropriate to engage in food preparation-related activities [30–33]. As a result, women are likely to be better trained to economize food purchases, which might make the monthly allowance last longer and limit their vulnerability to food insecurity compared to men. In addition, a higher percentage of the men lived alone, without family or children. Previous studies have indicated that the waiting time at the reception centers, leads to deterioration of daily rhythms, with people tending to sleep or stay in their rooms until late in the day [12, 34, 35]. To be part of a family and having children—some of them attending school or kindergarten—might contribute to a more stable organization of life and family meals at asylum reception centers. Asylum seekers receive an allowance per person in the household and more women lived with children than men. Living with more children therefore results in a higher total monthly budget, which could be the explanation why those with more children had lower odds of experiencing adult food insecurity.

In our study, length of stay in Norway was not associated with adult food insecurity among asylum seekers, meaning that the risk of food insecurity did not diminish with duration of residence in Norway. Hadley, Anderson, et al. reported the same finding among Sudanese immigrants in the US, and suggested that the underlying reason for this persistent vulnerability was continued low employment and income due to language barriers, as well as low education and limited access to employment opportunities among this group of refugees [17, 27]. In Norway, only asylum seekers with an approved application have permission to work. Surprisingly we found no difference in monthly budget among asylum seekers with approved or rejected applications. This could be due to the difficulties of finding a job while still residing in asylum centers, often located in areas with limited work opportunities, or by the fact that the asylum seekers also have some form of additional economic support from relatives and friends. However this was not a topic specifically investigated in our study.

The energy and nutrient requirements of children (especially young children and adolescents) to maintain good health and secure optimal development are high [36]. In order to cover their micronutrient needs, children (to a larger extent than adults) must rely on foods with high nutrient density, which are generally more costly. Household food insecurity has been associated with insufficient energy intake and child hunger [37], as well as a reduced quality of diet in different settings [38, 39]. Since their requirements are higher [36], the negative health effects of insufficient food and poor quality diets will be noticeable earlier in children than in adults. Thus, within the food insecurity scale, child hunger is the most serious category, wherein caregivers report that they know the child is sometimes hungry but cannot afford to buy more food. The present study found that 20% of the families had experienced child hunger. In a study of recently resettled Sudanese refugees in the US [17], 14% reported child hunger, and among Liberian refugees in the US [4], 42% experienced severe levels of food insecurity or child hunger. Additionally, a UK study reported that 60% of the children in refugee households were experiencing hunger [18], and recent arrival, being a younger mother, and lack of access to benefits are risk factors for child hunger. Maternal education was not a risk factor for child hunger. Conversely, length of stay in the US, maternal education, income, and being employed were negatively associated with child hunger among Liberian refugees in the US [4].

The primary strength of this study is the relatively high number of participants, including men and women of different nationalities, which provides novel and broad knowledge on the extent of food insecurity and some of its determinants in a European country. It is an especially important study as there has been little research investigating food security in Europe. Sellen assessed food security among refugees in London with the Radimer/Cornell Scale in 2002 [18]. Different ways of measuring self-reported food security can be found in the literature [40–42]. According to a systematic review of methods to measure food security [40], the instruments most frequently found in the scientific literature were the Household Food Security Survey Module Six-Item Short Form, the Self-Perceived Household Food Security Scale and the Radimer/Cornell Scale. Key strengths of the Radimer/Cornell scale is that it is well-grounded conceptually, being based on an in-depth understanding of the experience of food insecurity in the households. Each set of the food insecurity and hunger questions (Table 2) captures a different degree of severity, and the full range of severity and distinguishes among its different levels. This feature is critical for accurately gauging the prevalence of each level of severity. In addition, the Radimer/Cornell scale is quick and simple to administer. The limitations of the scale include the determination of cutoff points for defining food insecurity, mitigating potential response bias from experience-based measures and actual measurement of dietary adequacy [41, 43]. Other methods for assessing food security can be used, such as the 24-h dietary recall or food frequency recall data. While these methods might have the advantage of providing more reliable assessment, they still present measurement challenges. For example, how many and which food groups to include in the measure, how to account for the quantity of each food group consumed, what recall period to use, and how to assign cutoff values for defining levels of dietary diversity [43]. Therefore, the use of complementary approaches could contribute to ameliorating the assessment of food security.

There are also some limitations to the study. First, the convenience sampling strategy used to identify the participants precludes generalization of the findings to all asylum seekers in Norway. The findings remain valid for the participants involved and may well be indicative of conditions experienced by other asylum seekers. Second, the sample size was relatively small in terms of the group of families who experienced child hunger (8 out of 41 families). Consequently we did associational analyses only evaluating adult food insecurity with hunger and no other food insecure categories. Third, the study was cross-sectional and only assessed bivariate associations. Consequently, we are limited in our ability to determine the direction of associations with food insecurity and cannot fully disentangle relationships between the socioeconomic variables themselves. Food insecurity is a complex phenomenon, with a number of factors at play [40, 41]. Food security measures based on an individual’s self-reported experiences need to take into consideration contextual factors that might influence data collection. In our study, some of the reception centers received news that they were going to close down while we were collecting data. This caused uncertainty and frustration among the residents, as they were going to be relocated to other centers, which could have influenced their responses to the questionnaire.

## Conclusions

This study, which was the first of its kind on food security among asylum seekers in Norway, found a high prevalence of food insecurity with hunger among adults and children. The asylum seekers in the reception centers were worried about having enough money to buy food, they limited their dietary intake in order to stretch the budget, experienced being hungry and in some situations not having enough food for their children. The seriousness of this problem has to be considered in light of the affluence of the Norwegian society. Therefore, initiatives to promote adequate diet are of the outmost importance in this population. Immigration authorities and volunteer organizations involved in assisting asylum seekers should provide programs to ameliorate the opportunities for an adequate diet.
